# An endophytic *Schizophyllum commune* possessing antioxidant activity exhibits genoprotective and organprotective effects in fresh water fish *Channa punctatus* exposed to bisphenol A

**DOI:** 10.1186/s12866-022-02713-9

**Published:** 2022-12-06

**Authors:** Avinash Sharma, Pooja Chauhan, Khushboo Sharma, Vishali Kalotra, Anupam Kaur, Pooja Chadha, Sukhraj Kaur, Amarjeet Kaur

**Affiliations:** 1 Department of Microbiology, Guru Nanak Dev University, 143005 Amritsar, Punjab India; 2 Department of Zoology, Guru Nanak Dev University, 143005 Amritsar, Punjab India; 3 Department of Human Genetics, Guru Nanak Dev University, Amritsar, Punjab India

**Keywords:** Antioxidant, Basidiomycetes, Endophyte, Genoprotective, Organprotective, *Schizophyllum commune*

## Abstract

**Background:**

Oxidative stress is responsible for the onset of several chronic and degenerative diseases. Exogenous supply of antioxidants is reported to neutralize the effects of oxidative stress. Several synthetic antioxidants suffer from various side effects which necessitates the exploration of antioxidant compounds from natural sources. Endophytic fungi residing in the plants are gaining the attention of researchers as a source of novel antioxidants. Majority of the research conducted so far on endophytic fungi has been restricted to the members of phylum ascomycota. Basidiomycota, inspite of their immense bioactive potential remain relatively unexploited. This study aimed to assess the ameliorative effects of an endophytic *Schizophyllum commune* (basidiomycetous fungus) against oxidative stress associated altered antioxidant levels, genotoxicity and cellular damage to different organs in bisphenol A exposed fresh water fish *Channa punctatus*.

**Results:**

Good antioxidant and genoprotective potential was exhibited by *S. commune* extract in *in vitro* studies conducted using different antioxidant, DNA damage protection, and cytokinesis blocked micronuclei assays. In vivo studies were performed in fresh water fish *Channa punctatus* exposed to bisphenol A. A significant decrease in the considered parameters for DNA damage (% micronuclei and comet assay) were recorded in fish treated with *S. commune* extract on comparison with untreated bisphenol A exposed group. The *S. commune* extract treated fish also exhibited an increase in the level of antioxidant enzymes *viz*. catalase, superoxide dismutase and glutathione reductase as well as histoprotective effect on various organs. GC-MS analysis revealed the presence of 3-n-propyl-2,4-pentanedione, n-heptadecanol-1, trans-geranylgeraniol, 3-ethyl-2-pentadecanone, 1-heneicosanol and squalene as some of the compounds in *S. commune* extract.

**Conclusion:**

The study highlights the significance of an endophytic basidiomycetous fungus *S. commune* as a source of antioxidant compounds with possible therapeutic potential.

**Supplementary Information:**

The online version contains supplementary material available at 10.1186/s12866-022-02713-9.

## Background

Oxidative stress resulting from the unregulated production of reactive oxygen species (ROS) and reactive nitrogen species (RNS) plays a crucial role in the etiology of several chronic and degenerative diseases [1–3] and has also been reported to cause histopathological alterations in different organs [4]. One of the major outcomes of oxidative stress is DNA damage which plays a significantly important role in various diseases like cancer, Wilson’s disease and age-related neurodegenerative disorders [5–8]. In response to oxidative stress, different endogenous enzymatic and non-enzymatic antioxidant mechanisms are present in the human body which play a crucial role in the neutralization and detoxification of the oxidative stress damage. An exogenous supply of antioxidants could help the endogenous mechanisms to neutralize oxidative stress. Exogenous antioxidants like carotenoids, vitamin C, vitamin E, omega-3 fatty acids, omega-6 fatty acids, trace metals and flavonoids etc. can be received through diet and supplements [1, 9].

Antioxidants provide protection through different mechanisms, including transformation of ROS into non-radical species, reducing localized oxygen concentrations and breaking ROS initiated auto-oxidative chain reactions [10]. Compounds exhibiting antioxidant potential have been reported to protect DNA from oxidative stress induced damage [11, 12]. Recently the interest of scientific research fraternities has increased markedly for the exploration of antioxidants from natural sources. A variety of plants and endophytic fungi have been reported to exhibit antioxidant activities [10, 13, 14]. Endophytes are the microorganisms which inhabit the living plant tissues for variable periods of their life cycle without causing any symptomatic infection [15]. Compounds produced by endophytic fungi have been reported to possess anticancer, antidiabetic, antioxidant, antiviral, antiparasitic, antibacterial and immunomodulatory activities [14, 16]. In literature majority of the bioactive metabolites have been reported from endophytic fungi belonging to phylum ascomycota. Endophytic basidiomycetes have not been exploited extensively for their bioactive potential, despite having the ability to produce compounds exhibiting application in different fields such as agriculture, medicine and industry [17–19]. Considering the unexplored potential of endophytic basidiomycetes, in previous studies conducted in our lab we have made an attempt to isolate endophytic basidiomyctes and assess them for various bioactivities. An isolate Sch1, identified to be *Schizophyllum commune* was isolated from *Aloe vera* [20] and used in the present study. *S. commune* (Sch1), also known as split gill mushroom, belongs to class agaricomycetes of phylum basidiomycota. It is an edible mushroom and being consumed in several regions of the world especially in Mexico, Nagaland (India) and Republic of the Congo [21–23]. *S. commune* has been reported to possess antioxidant potential [24–26], but its genoprotective and organprotective potential under the influence of oxidative stress has not been studied. So in this study in vivo genoprotective, and organprotective effects of the endophytic *S. commune* (Sch1), procured from the culture collection of our lab were assessed after preliminary screening of its antioxidant potential. In vivo protective effects were determined in bisphenol A (BPA) exposed fish *Channa punctatus*.

## Results

**Fig. 1:**
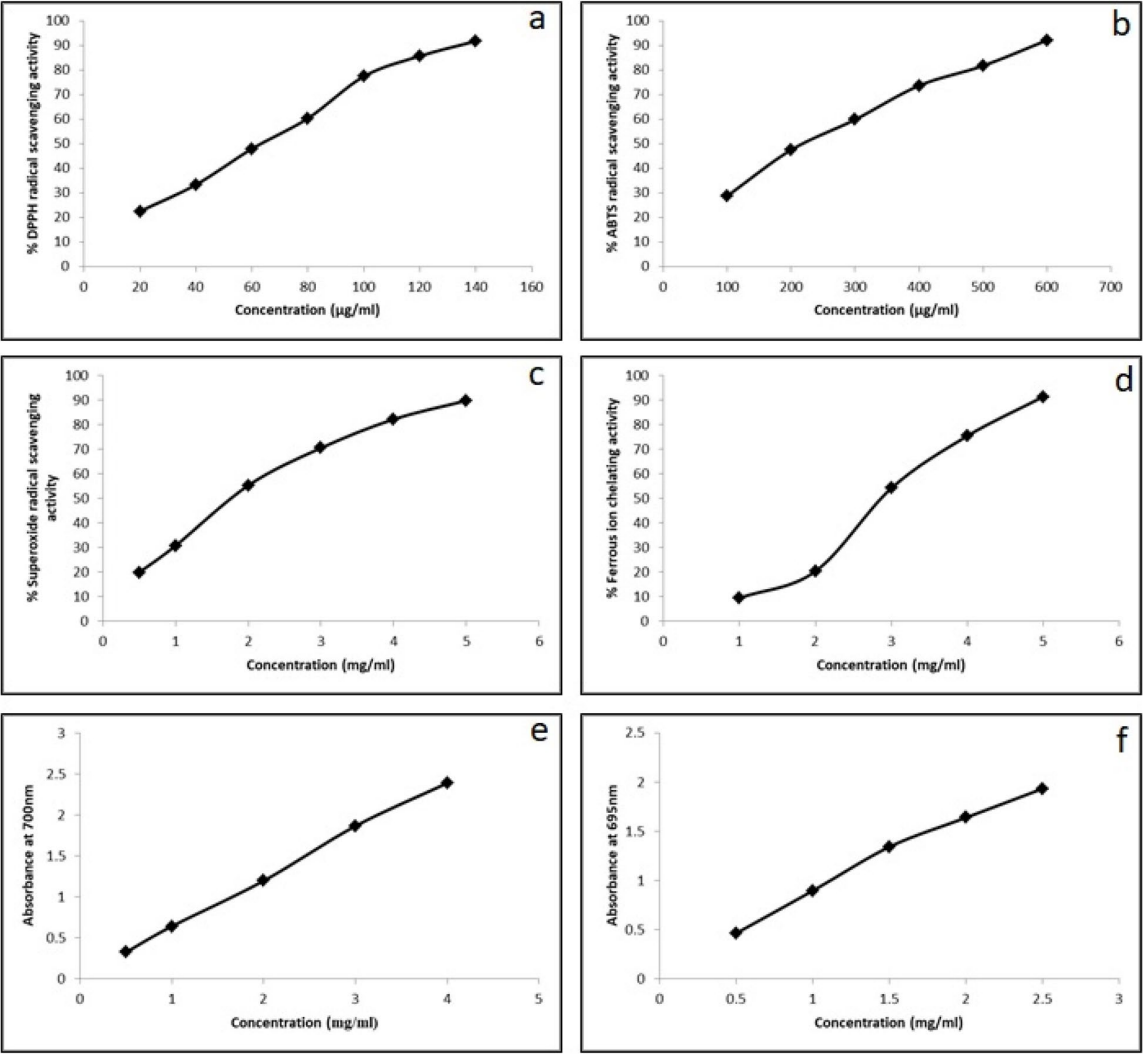
Antioxidant potential of *S. commune* (Sch1) extract (**a**) DPPH radical scavenging activity, (**b**) ABTS radical scavenging activity, (**c**) superoxide radical scavenging activity, (**d**) ferrous ion chelating activity, (**e**) FRAP, (**f**) total antioxidant capacity

In the present study, an endophytic culture of *Schizophyllum commune* (Sch1) obtained from the culture collection of our lab was evaluated for antioxidant and genoprotective potential. *In vitro* antioxidant potential was assessed by using various assays *viz*. 2,2-diphenyl-1-picrylhydrazyl (DPPH) radical scavenging, 2,2’-azino-bis (3-ethylbenzthiazoline-6-sulphonic acid) (ABTS) radical scavenging, superoxide radical scavenging, ferrous ion chelating, ferric reducing antioxidant power (FRAP) and total antioxidant capacity assays. The *S. commune* (Sch1) extract exhibited strong activities in DPPH, ABTS and superoxide radical scavenging assays. IC_50_ values of *S. commune* (Sch1) extract for DPPH, ABTS, and superoxide radical scavenging activities were calculated to be 50.61 µg/ml, 198.042 µg/ml, and 1.503 mg/ml, respectively. The radical scavenging activity of the extract increased with increase in concentration indicating a dose dependent effect as depicted in Fig. 1a, b and c. The antioxidant potential of *S. commune* (Sch1) extract was also revealed by ferrous ion chelating activity where antioxidant compounds present in the extract caused a dose dependent decrease in the absorbance with IC_50_ value of 2.62 mg/ml (Fig. 1d). In FRAP assay, presence of antioxidant compounds causes transformation of yellow coloured test solution into various shades of blue and green corresponding to the reducing power of the extract. Increase in the absorbance because of the ferrous ion formation was measured at 700 nm. Total antioxidant capacity of *S. commune* (Sch1) extract was also determined. *S. commune* extract revealed a concentration dependent reducing ability and total antioxidant capacity as evident in Fig. 1e,f. FRAP and total antioxidant capacity of *S. commune* (Sch1) extract were expressed as ascorbic acid equivalents (AAE)/mg of extract and quantified to be 72.25 and 249.83 µg AAE/mg of extract, respectively.

### *In vitro *genoprotective study

*In vitro* assessment of genoprotective potential of *S. commune* (Sch1) was performed by using DNA damage protection assay and cytokinesis blocked micronuclei (CBMN) assay.

### DNA protection assay

**Fig. 2:**
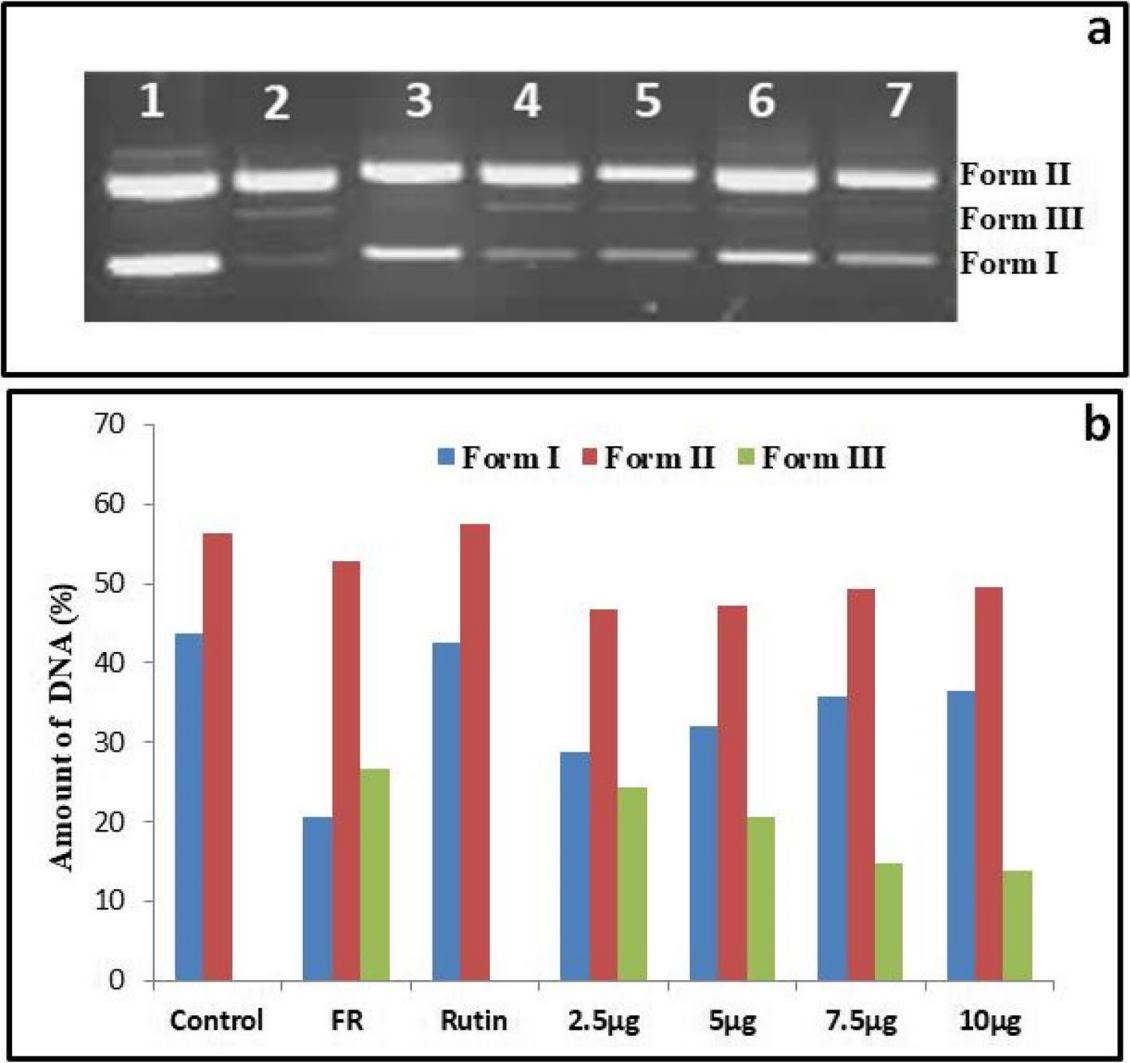
**a** DNA protection potential of *S. commune* (Sch1) extract. Lane 1: plasmid DNA; lane 2: plasmid DNA + Fenton’s reagent; Lane 3: plasmid DNA + Fenton reagent + rutin (positive control); Lane 4–7: plasmid DNA + Fenton reagent + different concentrations of *S. commune* (Sch1) extract (2.5, 5, 7.5, 10 µg); **b** Densitometric analysis of different forms of plasmid: control (plasmid DNA); FR (plasmid DNA + Fenton’s reagent); Rutin (plasmid DNA + Fenton reagent + rutin); 2.5-10 µg (plasmid DNA + Fenton reagent + different concentrations of *S. commune* (Sch1) extract). Form I- supercoiled DNA; Form II- nicked (open circle) DNA; Form III- linear DNA

In the presence of Fenton reagent, supercoiled form of plasmid DNA cleaves into linear or open circular form. Addition of different concentrations of *S. commune* (Sch1) extract protected the supercoiled form of plasmid DNA (Fig. 2a). Densitometric analysis of different forms of pBR322 plasmid DNA was done by using ImageJ software. As displayed in Fig. 2b, exposure to Fenton reagent was recorded to cause a reduction in form I (supercoiled) from 43.65% to 20.5% when compared with normal control. At different concentrations of *S. commune* (Sch1) extract protection of supercoiled plasmid DNA was observed as it was found to be 28.89 %, 32.12%, 35.85 % and 36.49% in the presence of 2.5 µg, 5 µg, 7.5µg and 10 µg of *S. commune* extract, respectively.

### CBMN assay

**Fig. 3:**
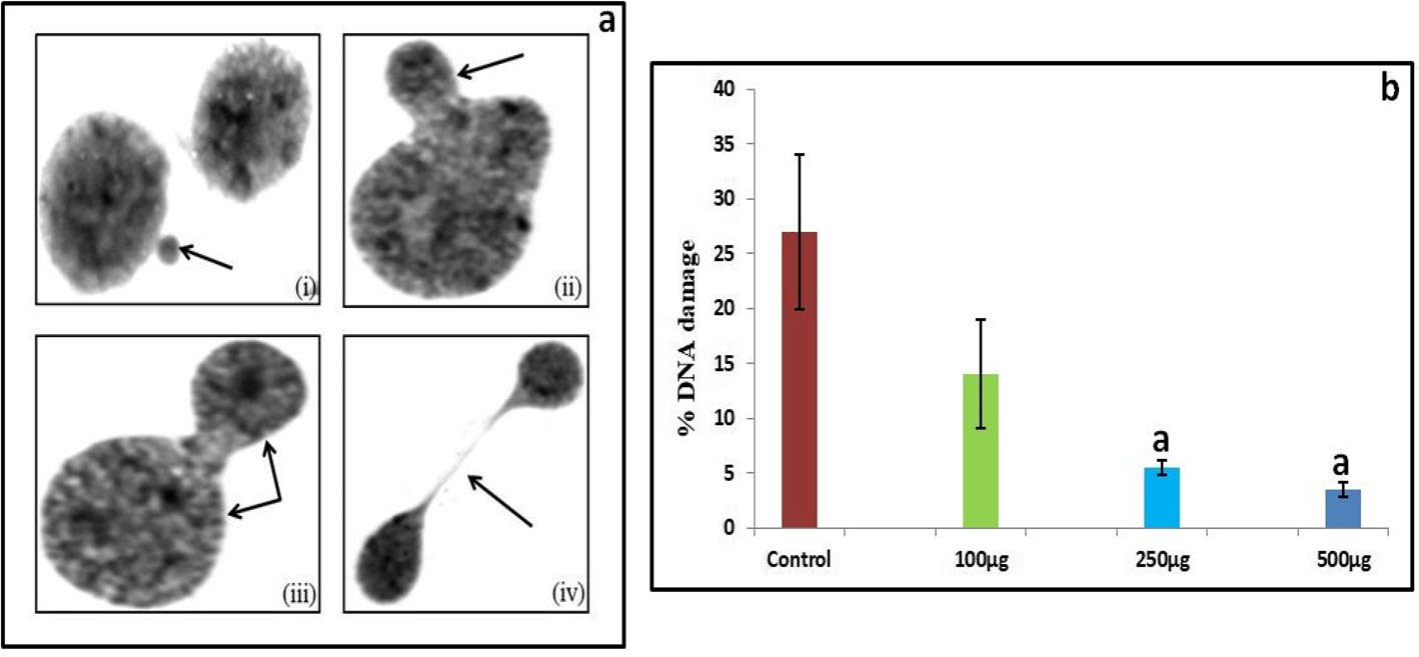
**a** Different forms of DNA damage observed (i) micronuclei (ii) nuclear bud (iii) binucleated cell and (iv) nucleoplasmic bridge in lymphocytes exposed to H_2_O_2_; **b** % DNA damage in human lymphocytes; control (250µM H_2_O_2_); 100 µg (250µM H_2_O_2_ + 100 µg/ml *S. commune* (Sch1) extract); 250 µg (250µM H_2_O_2_ + 250 µg/ml *S. commune* (Sch1) extract); 500 µg (250µM H_2_O_2_ + 500 µg/ml *S. commune* (Sch1) extract). ^a^P< 0.05 Different treated groups when compared with control (H_2_O_2_) group

DNA damage was measured in the form of micronuclei, nucleoplasmic bridge, nuclear buds and binucleated cells (Fig. 3a). Total % DNA damage caused by H_2_O_2_ in lymphocytes was found to be 27 ± 7.07. Total % DNA damage after treatment with 250 µg/ml and 500 µg/ml *S. commune* (Sch1) extract was recorded to be 5.5 ± 0.71 and 3.5 ± 0.71, respectively, indicating a significant (*p* < 0.05) decrease when compared with control (Fig. 3b). Better protective effect was observed at highest concentration of *S. commune* (Sch1) extract. On the other hand, no DNA damage was observed in group which contained only human lymphocytes.

### *In vivo* genoprotective study

#### Micronuclei assay

**Fig. 4:**
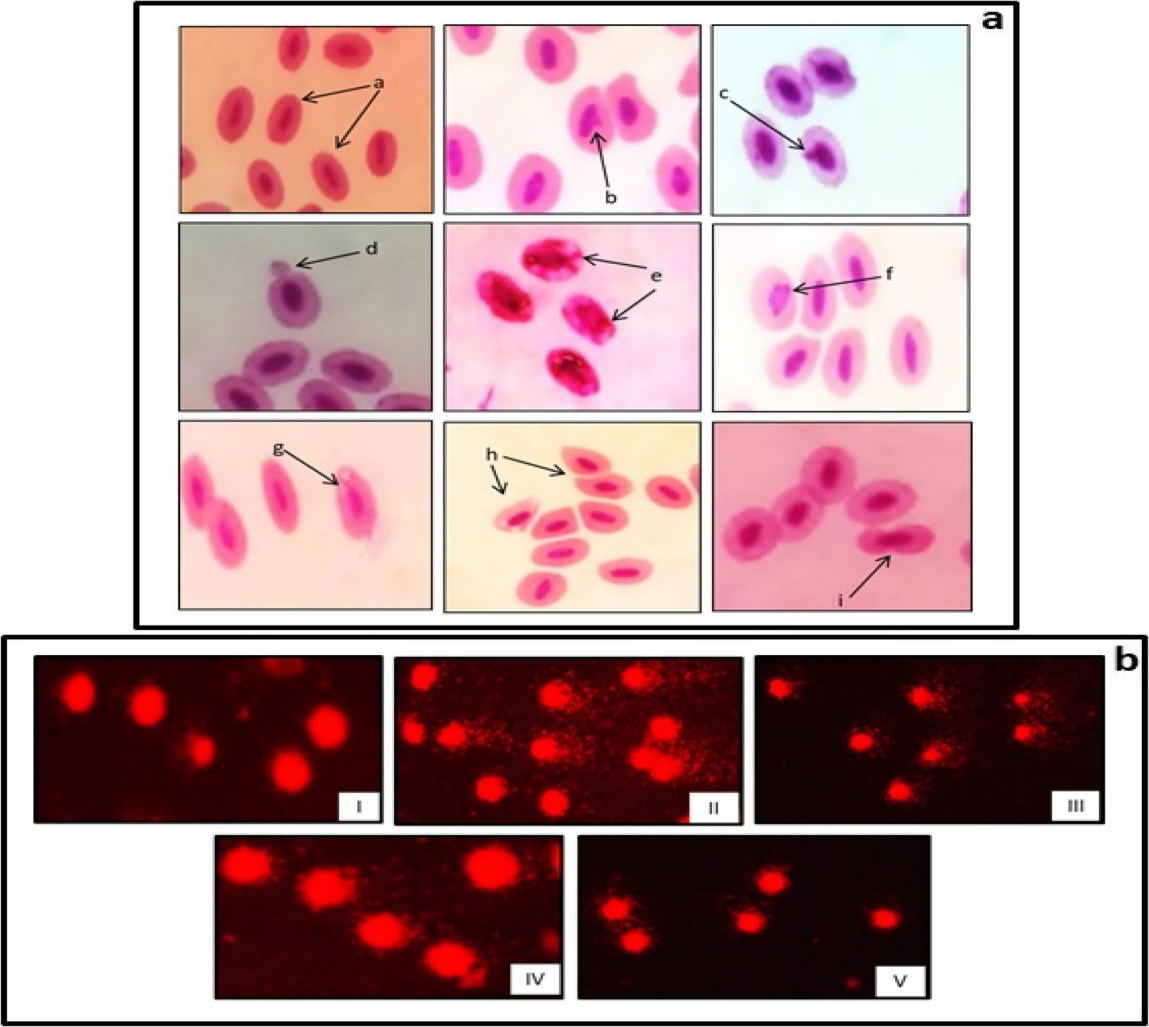
**a** Microscopic images of blood cells of *C. punctatus* showing (a) normal erythrocytes, (b) lobed nuclei, (c) micronuclei, (d) cytoplasmic bulge, (e) caryolised cell, (f) notched nucleus, (g) vacuolated cell, (h, i) other deformities; **b** fluorescent microscopic images showing different degree of DNA damage (I) normal healthy control, (II, III) cells from fish exposed to BPA, (IV, V) cells from fish treated with *S. commune* (Sch1) extract

**Fig. 5:**
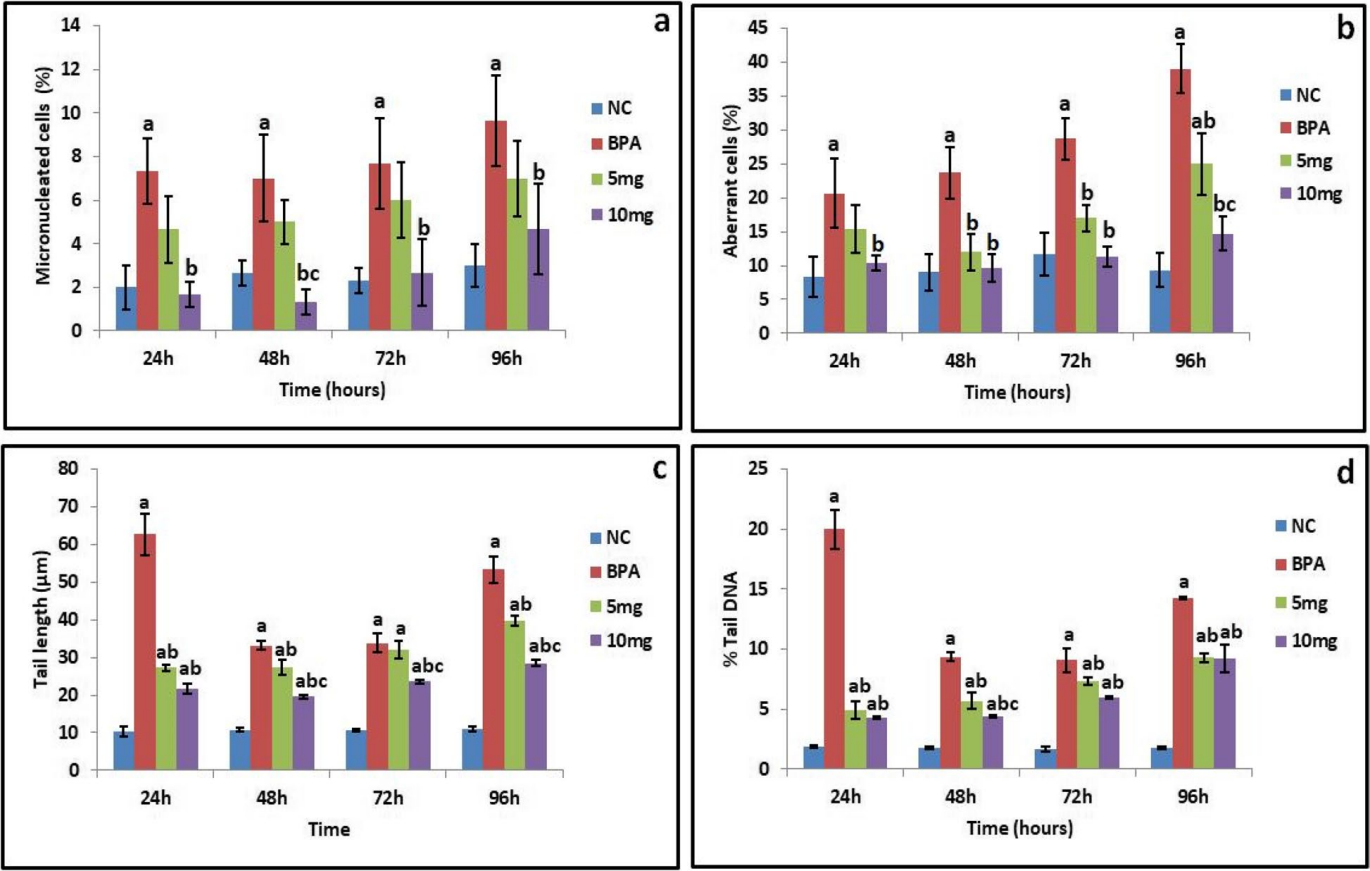
Effect of *S. commune* (Sch1) extract on **a** micronucleated cell frequency (%); **b** aberrant cell frequency (%); **c** tail length; **d** % tail DNA in the blood cells of *C. punctatus* at different time intervals. Data are expressed as mean ± S.D. (*n* = 3). NC: normal healthy control; BPA: fish exposed to BPA; 5 mg and 10 mg: BPA exposed fish treated with *S. commune* (Sch1) extract (5 mg and 10 mg/100 g body weight, respectively). ^a^P< 0.05 Different groups when compared with normal control group. ^b^P< 0.05 BPA exposed group when compared with treated groups. ^c^P< 0.05 *S. commune* (Sch1) extract treated groups when compared with each other

Fish exposed to sublethal concentration of BPA were administered with *S. commune* (Sch1) extract (5 mg/100 g and 10 mg/100 g body weight) to determine its effect on micronuclei and aberrant cell frequency. Micronuclei and other aberrant cells (notched, fused cells, lobed, vacuolated cells, cytoplasmic bulge and caryolised cells) (Fig. 4a) frequency was observed in erythrocytes of fish at different time intervals. Treatment with higher concentration of *S. commune* (Sch1) extract showed significant (*p* < 0.05) decrease in percent micronuclei frequency (Fig. 5a). Maximum decrease of 81% was observed after 48 h in fish treated with higher concentration as compared to BPA exposed untreated group.

Aberrant cell frequency also significantly (*p* < 0.05) decreased in fish treated with higher concentration of *S. commune* (Sch1) extract as shown in Fig. 5b. On comparison with BPA exposed untreated fish, maximum percent decrease of 62.4 (after 96 h) in aberrant cell frequency was observed in fish treated with 10 mg of *S. commune* (Sch1) extract.

#### Comet assay

Tail length and % tail DNA were used as standard parameters to assess the protective effect of *S. commune* extract on DNA damage. After 24 h, tail length and % tail DNA were significantly (*p* < 0.05) increased in the blood cells of the fish exposed to BPA when compared with normal healthy control group. Treatment with both the concentrations of *S. commune* (Sch1) extract significantly (*p* < 0.05) reduced the tail length and % tail DNA as compared to BPA exposed untreated group at most of the time intervals (Figs. 4b and 5c, d). Throughout the experimental period higher concentration of *S. commune* (Sch1) extract protected the DNA from damage as the level of considered parameters remained significantly (*p* < 0.05) lowered as compared to untreated BPA exposed group. The maximum effect of both the concentrations of *S. commune* (Sch1) extract was recorded after 24 h. In case of lower concentration a percent decrease of 56.5 and 75.2 in tail length and % tail DNA, respectively, was observed after 24 h as compared to untreated BPA exposed group. Treatment with higher concentration of *S. commune* (Sch1) extract showed 65.5 and 78.7% decrease in tail length and % tail DNA, respectively, after 24 h on comparison with untreated BPA exposed fish. As evident from Fig. 5c, d, higher concentration of *S. commune* (Sch1) extract was found to be more effective.

### Antioxidant enzymes

#### Catalase (CAT) estimation

**Fig. 6:**
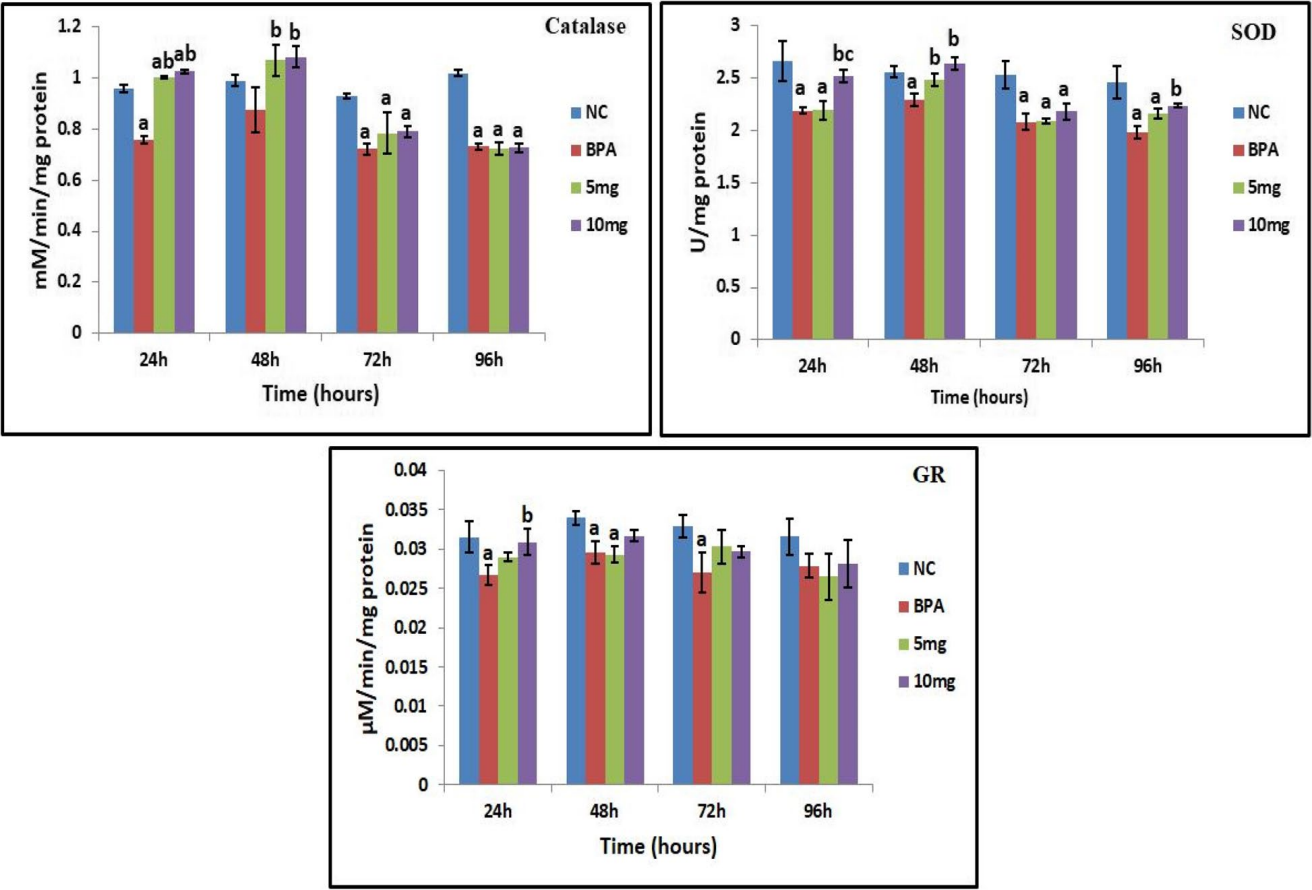
Effect of *S. commune* (Sch1) extract on catalase, SOD, and GR levels in BPA exposed fish. Data are expressed as mean ± S.D. (*n* = 3). NC: normal healthy control; BPA: fish exposed to BPA; 5 mg and 10 mg: BPA exposed fish treated with *S. commune* (Sch1) extract (5 mg and 10 mg/100 g body weight, respectively). ^a^*P*< 0.05 Different groups when compared with normal control group. ^b^*P*< 0.05 BPA exposed group when compared with treated groups. ^c^P< 0.05 *S. commune* (Sch1) extract treated groups when compared with each other

Activity of catalase in fish exposed to BPA was found to be decreased significantly (*p* < 0.05) on comparison with normal healthy control group at most of the time intervals. Treatment with *S. commune* (Sch1) extract significantly (*p* < 0.05) increased the catalase activity in fish exposed to BPA after 24 and 48 h when compared with BPA exposed untreated group. Maximum effect of *S. commune* (Sch1) extract was observed after 24 h as increase of 32.4 and 35.2% in catalase activity was found in groups treated with lower (5 mg) and higher (10 mg) concentrations, respectively, when compared with BPA exposed untreated group (Fig. 6).

#### Superoxide dismutase (SOD) estimation

Level of SOD was found to be significantly (*p* < 0.05) reduced in BPA exposed group when compared with normal healthy control group. As shown in Fig. 6, group treated with higher concentration of *S. commune* (Sch1) extract showed significant (*p* < 0.05) increase in SOD level after 24 and 48 h with activity of 2.515 ± 0.061 U/mg protein and 2.634 ± 0.059 U/mg protein, respectively, as compared to BPA exposed untreated group. Results obtained were more pronounced at higher concentration of *S. commune* (Sch1) extract.

#### Glutathione reductase (GR) estimation

Level of GR was found to be significantly (*p* < 0.05) reduced in the fish exposed to BPA when compared with normal healthy control group at most of the time intervals. Maximum effect was observed after 24 h as significant (*p* < 0.05) increase of 15.7% was recorded in BPA exposed group treated with higher concentration of *S. commune* (Sch1) extract when compared with untreated BPA exposed control group (Fig. 6).

#### Histopathological study

**Fig. 7:**
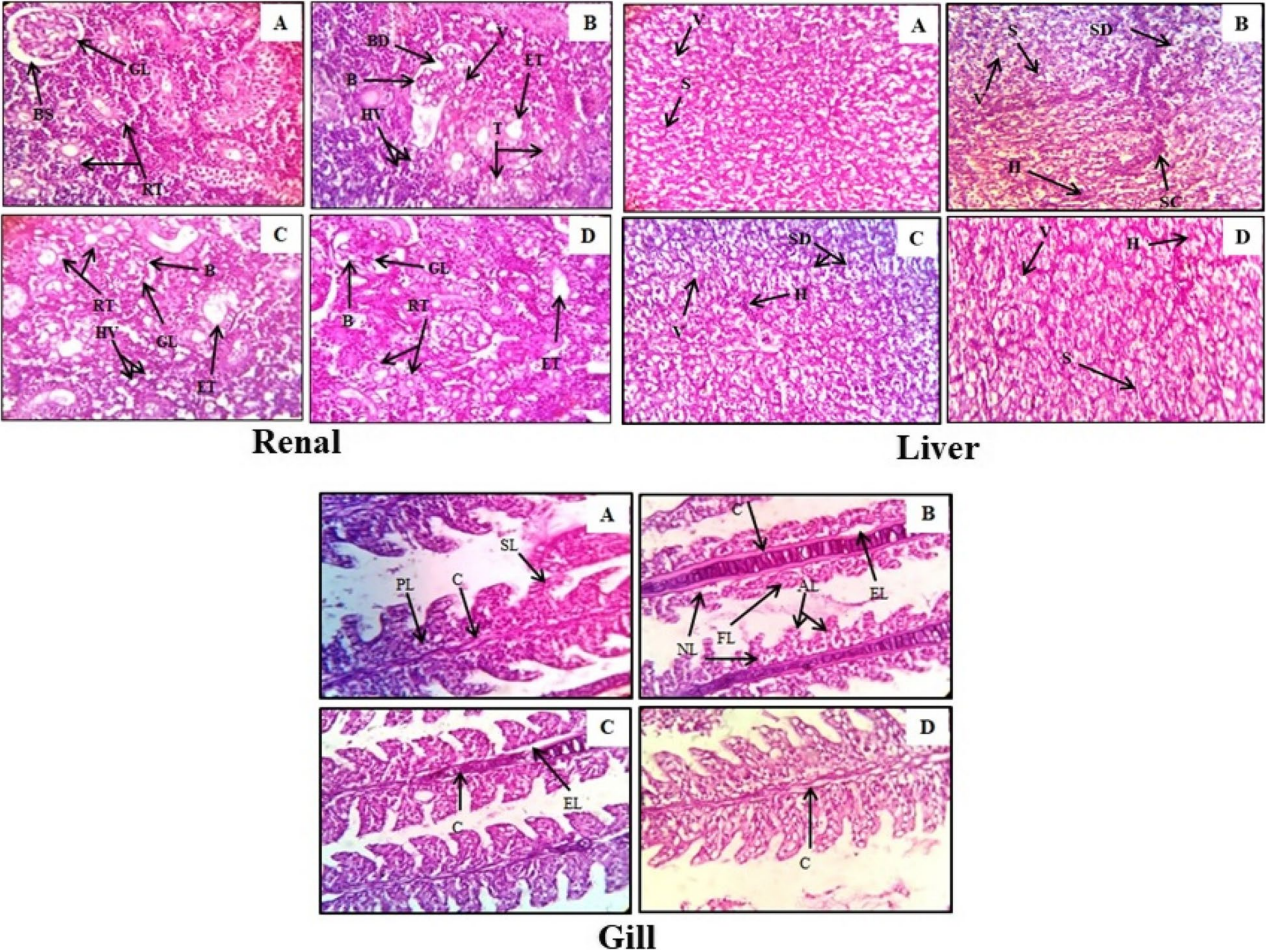
Photomicrographs of H & E-stained renal, liver and gill tissue of *C. punctatus* at 40x (**A**) normal control, (**B**) fish exposed to BPA, (**C**) fish treated with lower concentration of *S. commune* (Sch1) extract (5 mg), (**D**) fish treated with higher concentration of *S. commune* (Sch1) extract (10 mg). Renal: (GL) Glomerulus, (BS) well defined Bowman’s space, (RT) renal tubules, (BD) Bowman’s space dilation, (**B**) disorganized blood capillaries, (HV) vacuolization of hematopoietic tissue, (V) vacuolization in glomerulus, (ET) enlargement of renal tubule (T) loss of cellular integrity of tubular cells; Liver: (S) Sinusoids, (V) cytoplasmic vacuolization, (H) hepatocyte atrophy, (SD) dilation of sinusoids, (SC) sinusoids congestion; Gill: (SL) Secondary lamellae, (**C**) cartilage, (PL) primary lamellae, (EL) epithelial lifting, (NL) necrosis in lamellae, (FL) fusion of lamellae, (AL) abnormal secondary lamellae

Histopathological effects of *S. commune* (Sch1) extract were examined in liver, kidney and gill of *C. punctatus.* Hepatic tissue of BPA exposed untreated fish displayed structural alterations including cytoplasmic vacuolation, hepatocytic atrophy with increased dilation of sinusoids and sinusoids congestion. The groups treated with *S. commune* (Sch1) extract showed noteworthy better structural architecture in comparison with BPA exposed untreated group (Fig. 7).

Histopathological study of renal tissue showed well defined Bowman’s space with normal structure of glomerulus and renal tubules in normal control group. In case of BPA exposed group Bowman’s space dilated with disorganised blood capillaries, vacuoles in hematopoietic tissue and glomerulus were also observed. Renal tubules were also enlarged and lost their cellular integrity to some degree. All these structural deformities were found to be decreased in *S. commune* (Sch1) extract treated groups (Fig. 7).

Different histopathological alterations were also observed in gill of fish exposed to BPA. Epithelial lifting, fusion of secondary lamellae, abnormally shaped secondary lamellae and some degree of necrosis was recorded in BPA exposed untreated group. Fish treated with *S. commune* (Sch1) extract displayed better structural architecture of gill as compared to BPA exposed group (Fig. 7).

#### GC-MS analysis

**Fig. 8:**
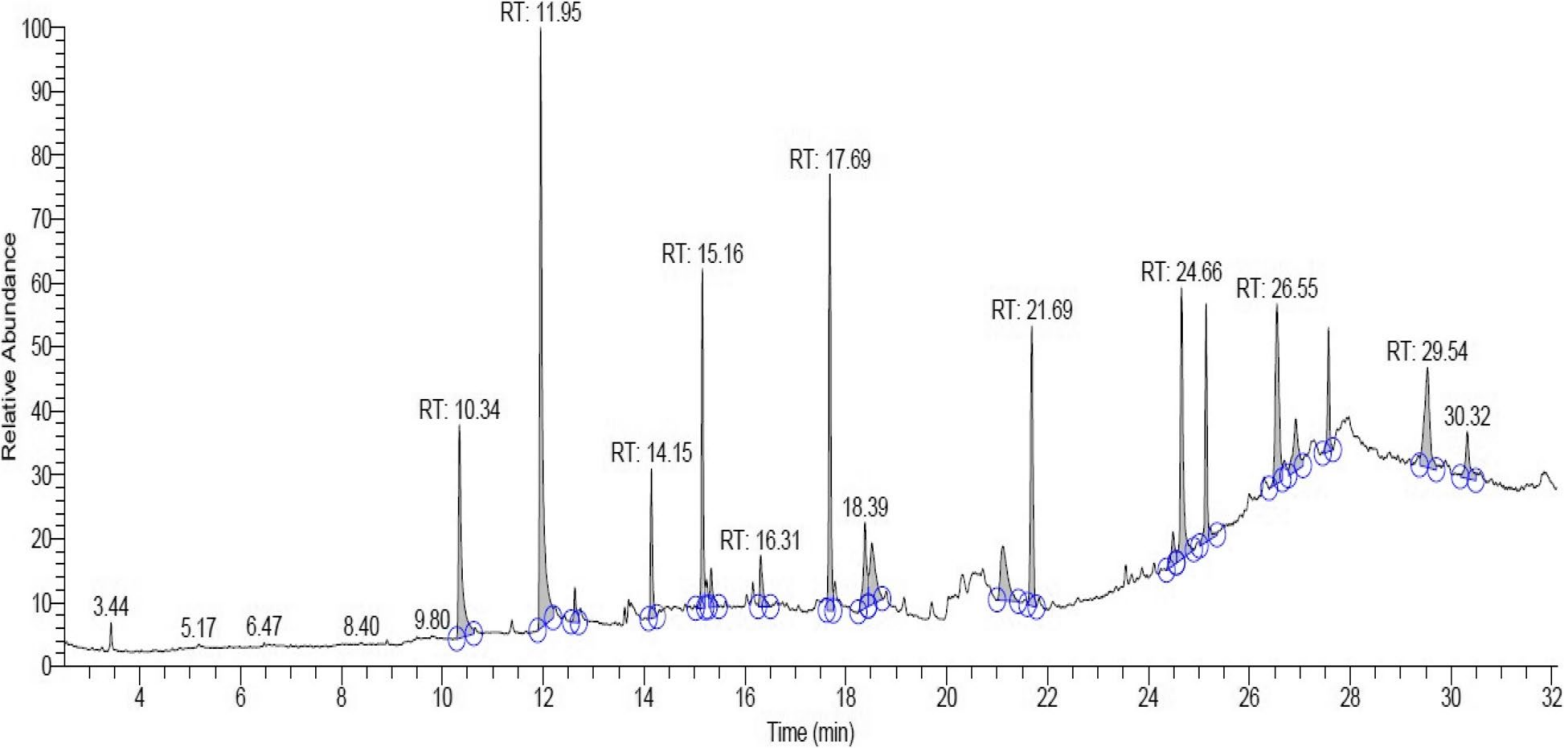
GC-MS analysis of *S. commune* (Sch1) extract

GC-MS analysis of *S. commune* (Sch1) extract revealed major compounds in the extract to be 3-n-propyl-2,4-pentanedione, n-heptadecanol-1, trans-geranylgeraniol, 3-ethyl-2-pentadecanone, 1-heneicosanol, and squalene. Some other compounds identified in the extract were 1-hexadecanol, tetracosane, 1-heneicosyl formate, phenol, 2,4-bis (1,1dimethylethyl)-, phytol, 9-hexacosene, and 2-propenoic acid, pentadecyl ester (Fig. 8; Table 1).

**Table 1:** Major compounds present in *S. commune* (Sch1) extract identified by GC-MS analysis

Compound Name	RT	Area %	Molecular Formula
3-Ethyl-2-pentadecanone	10.34	7.31	C_17_H_34_O
3-n-Propyl-2,4-pentanedione	11.95	17.48	C_18_H_14_O_2_
Phenol, 2,4-bis (1,1dimethylethyl)-	14.15	3.09	C_14_H_22_O
1-Hexadecanol	15.16	6.36	C_16_H_34_O
2-Propenoic acid, pentadecyl ester	16.31	1.64	C_18_H_34_O_2_
n-Heptadecanol-1	17.69	9.05	C_17_H_36_O
Phytol	18.39	3.09	C_13_H_26_O
1-Heneicosanol	21.69	7.20	C_21_H_44_O
trans-Geranylgeraniol	24.66	8.20	C_20_H_34_O
1-Heneicosyl formate	25.15	5.37	C_22_H_44_O_2_
Squalene	26.55	7.02	C_30_H_5_O
Tetracosane	29.54	5.66	C_24_H_5_O
9-Hexacosene	30.32	2.06	C_26_H_52_

## Discussion

The present study demonstrates the genoprotective and organprotective potential of an endophytic *S. commune* (Sch1) possessing antioxidant activity. *S. commune*, also known as split gill mushroom belongs to phylum basidiomycota and is consumed in many parts of the world [21–23, 27]. Association of oxidative stress with various chronic and degenerative diseases necessitates the exploration of compounds possessing antioxidant and genoprotective potential. Investigation on *in vitro* antioxidant potential was conducted using various assays. Use of more than one assay to determine antioxidant potential is highly recommended as a particular assay can only evaluate antioxidant potential for its specific reaction system [28]. In this study, a total of six antioxidant assays with different reaction mechanisms were used to assess *in vitro* antioxidant potential of *S. commune* (Sch1) extract. DPPH assay is the most commonly used robust and simple method to assess the antioxidant potential of compounds. DPPH• radicals are stable radicals having a deep purple colour. The basic principle of this assay is to measure the efficacy of antioxidant compounds to reduce DPPH• radicals. The reducing ability (donation of hydrogen atom or electron) of the compounds is directly proportional to the decrease in purple colour [12, 29]. Similar to DPPH assay, ABTS radical (ABTS•^+^) reduction takes place in the presence of antioxidants which results in decolourization of bluish-green coloured ABTS•^+^ [30]. *S. commune* (Sch1) extract showed strong hydrogen donating ability in DPPH and ABTS radical scavenging assay. DPPH radical scavenging potential of *S. commune* has also been documented by other researchers but the reported IC_50_ values (0.145 mg/ml-0.8 mg/ml) were higher as compared to *S. commune* (Sch1) strain used in the present study [24, 25]. Besides this, *S. commune* (Sch1) extract also demonstrated a good potential to scavenge superoxide anion radicals (O_2_**•**^−^). These radicals are one of the most powerful reactive oxygen species produced when oxygen is absorbed by living cells. O_2_•^−^ transforms into other free radicals and reactive oxygen species including hydroxyl radical and hydrogen peroxide [31]. In ferrous ion chelating assay, Fe^2+^ ions react with ferrozine to form a stable red or dark purple coloured ferrous ion–ferrozine complex having maximum absorbance at 562 nm. The formation of ferrous ion–ferrozine complex is inhibited in the presence of chelator or antioxidant, resulting in decrease in the intensity of the red or dark purple colour [30]. *S. commune* (Sch1) extract was also recorded to possess good ferrous ion chelating, FRAP, and total antioxidant capacity potential. FRAP assay is based on the reduction of the Fe^3+^ to Fe^2+^ in the presence of antioxidant compounds which further react with ferric chloride to form a dark blue colour ferric ferrous complex [32]. In total antioxidant capacity assay reduction of Mo (VI) to Mo (V) takes place in the presence of antioxidant compounds which results in a green coloured phosphate/Mo (V) complex under acidic pH [33]. Reducing ability of the extract is directly proportional to increase in the absorbance.

DNA damage induced by oxidative stress is responsible for different types of diseases including cancer, Wilson’s disease and age-related neurodegenerative disorders [5–8]. Oxidative stress can also cause changes in various biochemical parameters and affect the functioning of various organs [34, 35]. As the selected fungus demonstrated high *in vitro* antioxidant potential, it was further subjected to investigate its genoprotective and organprotective effects. In this study, pBR322 plasmid DNA and lymphocytes were used to determine *in vitro* genoprotective potential of *S. commune* (Sch1) extract. pBR322 plasmid DNA was exposed to Fenton’s reagent. Hydroxyl radicals generated from the Fenton reaction cleave the supercoiled plasmid DNA into nicked linear or open circular form [36, 37]. *S. commune* (Sch1) extract showed effective protection in the native supercoiled form of DNA indicating its genoprotective effect. A significant decrease in % DNA damage was also observed in hydrogen peroxide exposed lymphocytes treated with different concentrations of *S. commune* (Sch1) extract. Similar genoprotective effects of fungi have also been documented by other researchers. *Penicillium oxalicum* isolated from *Citrus limon* possessing strong antioxidant potential was also reported to exhibit genoprotective activity against Fenton’s reagent exposed plasmid DNA and H_2_O_2_ induced DNA damage in human lymphocytes [11]. Živković et al. [38] demonstrated the *in vitro* genoprotective effect of *Agaricus blazei* against H_2_O_2_ induced DNA damage in human peripheral blood cells. In different studies, the oxidative stress based DNA damage was found to be diminished on exposure to extracts having antioxidant potential [12, 39].

In vivo protective effect of *S. commune* (Sch1) extract against oxidative stress was analysed in fresh water fish *C. punctatus.* Fish models have been used for investigating in vivo protective effects of various compounds. Amelioration of chromium and selenium induced oxidative stress and inflammation in fish administered with compounds and natural extracts possessing antioxidant activities has been reported by various researchers [40–42]. Singh et al. [43] and Kaur et al. [11] tested the in vivo genoprotecive efficacy of the endophytic *Cladosporium velox* and *P. oxalicum* (exhibiting good *in vitro* antioxidant activities) on freshwater fish *C. punctatus.* Polysaccharides from mushroom *Inonotus obliquus* have also been reported to exhibit genoprotective activity in embryonic zebrafish [44].

*Channa punctatus* was exposed to genotoxic compound BPA to induce oxidative stress. There are several studies which state that BPA is responsible for the generation of oxidative stress and also causes DNA damage [45–47]. Exposure to BPA results in decreased levels of antioxidant enzymes. A reduced level of SOD and CAT was reported by Sharma and Chadha [47] in BPA exposed *C. punctatus*. The level of SOD and CAT was also found to decrease significantly in BPA administered rats [48, 49]. SOD and CAT are first-line defence antioxidants. These enzymes respectively convert superoxide radicals and hydrogen peroxides into harmless molecules [50]. GR maintains the supply of reduced glutathione, which scavenges reactive oxygen and nitrogen species and helps to control redox homeostasis [51]. In this study, exposure to BPA was found to reduce the level of SOD, CAT, and GR in the untreated group. Treatment with *S. commune* (Sch1) extract effectively improved the level of these antioxidant enzymes. Sukalingam et al. [52] also reported reduction in the levels of antioxidant enzymes under oxidative stress which were found to be improved on administration with *Justicia tranquebariesis* extract possessing antioxidant potential in experimental animals.

Genoprotective effect of the *S. commune* (Sch1) extract was evaluated by using micronuclei and comet assay. A significant increase in the percentage of micronuclei and aberrant cell was observed in BPA exposed untreated group. Other workers have also reported increased number of micronuclei in BPA administered rats and mice [53, 54]. Groups treated with *S. commune* extract significantly reduced the incidences of micronuclei and aberrant cells. Another method used to study the genoprotective potential was comet assay. This assay, also known as a single-cell gel electrophoresis assay, is a quick and sensitive method for detecting DNA damage in individual cells and can be used in *in vitro* and in vivo studies. The degree of DNA strand breakage is measured by the extent of migration [55]. Parameters used to assess the genotoxic damage induced by genotoxicants included tail length and % tail DNA. The length of DNA indicates the distance travelled by the DNA out of the cell [56]. Percentage tail DNA signifies the percentage of DNA travelled from the head and is considered as one of the most popular and suitable parameter to assess DNA damage [57]. In this study, tail length and tail DNA were found to be increased in the BPA exposed untreated group as compared to the normal healthy control group. BPA has also been reported to cause DNA damage in RWPE-1 cells as the tail intensities were found to be increased significantly as compared to control [58]. Sharma and Chadha [47] also observed DNA damage in BPA exposed *C. punctatus.* In the present study, treatment with *S. commune* (Sch1) extract effectively protected DNA from damage caused by the genotoxic compound, BPA. Mechanisms involved in the protection of DNA and reduction in micronuclei and aberrant cell frequency are not fully understood but it could be due to the high antioxidant potential of *S. commune* (Sch1) extract.

Histopathological studies showed that the exposure to BPA remarkably changed the normal histoarchitecture of the liver, kidney and gill. BPA induced oxidative stress and histopathological alterations in the organs of the exposed animals has been reported by various workers [59–61]. Studies have revealed that oxidative stress plays a crucial role in the histopathological changes in different organs [4]. In this study, histopathological changes were also corroborated with the results of antioxidant enzyme assays and genoprotective assessment which showed that exposure to BPA significantly altered the cellular antioxidant level with increased genotoxicity. Administration of *S. commune* (Sch1) extract exhibited remarkable protection of the normal histopathological architecture of liver, kidney and gill. Other workers have also reported the organprotective effect of different plant extracts possessing antioxidant activity in experimental animals exposed to oxidative stress [52, 62].

The antioxidant potential of *S. commune* (Sch1) extract observed in this study was confirmed by the findings of GC-MS analysis as some of the identified compounds such as squalene, phytol, n-heptadecanol-1 are reported to possess antioxidant activity [63–65]. Although no reports are available on the assessment of their genoprotective and organprotective potential, these detected compounds by virtue of their antioxidant activities could be responsible for the genoprotective and organprotective effects.

## Conclusion

From the results obtained it can be concluded that the endophytic *S. commune* (Sch1) can show genoprotective, and organprotective effects by virtue of its antioxidant potential. It can also be exploited in the field of therapeutics as a source of different bioactive compounds. The findings also highlight the potential of endophytic basidiomycetes, which are relatively unexplored, as sources of novel compounds.

## Materials and methods

### Materials

Potato dextrose agar, malt extract, DPPH, ABTS, potassium persulphate, potassium ferricyanide, trichloroacetic acid, ferric chloride, nitro blue tetrazolium (NBT), phenazine methosulphate (PMS), ferrozine, cytochalasin-B, hydroxylamine hydrochloride, NADPH, and oxidized glutathione were purchased from Himedia, India. Ammonium molybdate was purchased from SRL, India. Bisphenol A was purchased from Sigma-Aldrich, St. Louis, USA. All other reagents used in this study were of analytical grade.

### Microorganism

The culture of *S. commune* (Sch1) used in this study was isolated from healthy asymptomatic plant of *Aloe vera* in previous study conducted in our lab [20].

### Production of bioactive metabolites

Purified culture of *S. commune* (Sch1) was freshly activated on potato dextrose agar plate. One mycelial plug of 8 mm was cut from the periphery of the growing culture and inoculated in 250 ml Erlenmeyer flask containing 50 ml malt extract broth supplemented with 2% malt extract, 0.1% peptone and 2% dextrose. The inoculated flasks were then incubated in rotary shaker at 180 rpm for 10 days at 30℃. Thereafter, extraction of the metabolites was done by using ethyl acetate at 120 rpm for 1.5 h twice at 40℃, followed by the collection of upper organic phase. Rotary evaporator (BUCHI) was used to concentrate the collected organic phase to dryness. The concentrated extract was used in the further study.

### Determination of *in vitro* antioxidant and genoprotective potential

#### Experimental design

*In vitro* antioxidant potential of *S. commune* (Sch1) extract was determined by using different assays including DPPH free radical scavenging assay, ABTS radical scavenging assay, superoxide radical scavenging assay, ferrous ion chelating assay, FRAP assay, and total antioxidant capacity. *In vitro* genoprotective ability of *S. commune* (Sch1) extract was assessed by using DNA damage protection assay and CBMN assay on lymphocytes.

#### DPPH free radical scavenging assay

Potential of *S. commune* (Sch1) extract to scavenge DPPH free radicals was determined by following standard protocol described by Blois [66] with slight modifications. In 96 well plate 100 µl DPPH solution (0.2 mM in methanol) was mixed with 100 µl extract (dissolved in methanol) at varying concentrations. In control 100 µl methanol was added in 100 µl DPPH. Thereafter, plate was incubated at 37℃ for 30 min and decrease in absorbance was recorded at 517 nm. L-ascorbic acid was used as positive control. The percentage radical scavenging activity was calculated by using following formula:

$$\mathrm{Radical}\;\mathrm{scavering}\;\mathrm{activity}\;\left(\%\right)\;=\frac{Ac\mathit-As}{Ac}\times100$$

*Ac* was the absorbance of control and *As* was the absorbance of the sample.

#### ABTS radical scavenging assay

This assay was performed as described by Re et al. and Mayur et al. [67, 68] with some modifications. ABTS radical cation (ABTS^•+^) was prepared by reacting equal volume of 7.4 mM ABTS with 2.6 mM potassium persulphate and allowed to react for 14–16 h at room temperature in dark. Before use, ABTS^•+^ solution was diluted with methanol to get an absorbance of 0.700 ± 0.020 at 734 nm. In reaction mixture 200 µl of diluted ABTS^•+^ solution was mixed with 10 µl extract at different concentrations and incubated for 6 min at room temperature. Absorbance was recorded at 734 nm using microtitre plate reader (FLUOstar Omega, BMG Labtech, Offenburg, Germany). The control consisted of 10 µl methanol instead of fungal extract. L-ascorbic acid was used as positive control. The percentage radical scavenging activity was calculated by using following formula:

$$\mathrm{Radical}\;\mathrm{scavenging}\;\mathrm{activity}\;\left(\%\right)\;=\;\frac{Ac\mathit-As}{Ac}\times100$$

*Ac* was the absorbance of control and *As* was the absorbance of the sample.

#### Superoxide radical scavenging assay

The superoxide radical scavenging potential of *S. commune* (Sch1) extract was investigated spectrophotometrically by measuring the reduced product of NBT [69]. All the reagents were prepared in phosphate buffer (100 mM, pH 7.4). Reaction mixture consisting of 1 ml *S. commune* (Sch1) extract at different concentrations, 1 ml PMS (60 μm), 1 ml NADH (468 μm), and 1 ml NBT (156 μm) was incubated at 25℃ for 5 min, and absorbance was read at 560 nm. Rutin was used as positive control. The % superoxide radical scavenging was calculated by using following formula.

$$\mathrm{Radical}\;\mathrm{scavenging}\;\mathrm{activity}\;\left(\%\right)\;=\;\frac{Ac\mathit-As}{Ac}\;\times100$$

*Ac* was the absorbance of control and *As* was the absorbance of the sample.

#### Ferrous ion chelating assay

Ferrous ion chelating activity of *S. commune* (Sch1) extract was determined according to the method of Mayur et al. [68]. In this assay, FeSo_4_ (2 mM) and ferrozine (5 mM) were diluted 20 times before use. FeSo_4_ (250 µl) was added to different concentrations of extract (250 µl) and reaction was initiated by adding 250 µl ferrozine. The reaction mixture was incubated at room temperature for 10 min and absorbance was taken at 562 nm. In control fungal extract was replaced by methanol. Ethylenediamine tetraacetic acid disodium (EDTA-Na_2_) was used as positive control. The % ferrous ion chelating activity of *S. commune* (Sch1) extract was calculated by using following formula.

$$\mathrm{Ferrous}\;\mathrm{ion}\;\mathrm{chelating}\;\mathrm{activity}\;\left(\%\right)\;=\;\frac{Ac-As}{Ac}\times100$$

*Ac* was the absorbance of control and *As* was the absorbance of the sample.

#### FRAP assay

The potential of *S. commune* (Sch1) extract to reduce ferric ions into ferrous was determined by the method of AK and Gülçin [32]. An aliquot (10 µl) of fungal extract was mixed with 15 µl sodium phosphate buffer (0.1 M, pH 7.6) followed by addition of 15 µl potassium ferricyanide (1% w/v). This reaction mixture was incubated for 20 min at 50℃ and acidified with 15 µl trichloroacetic acid (10%). Thereafter, 55 µl distilled water and 110 µl ferric chloride (0.1%) was added and the increase in absorbance was measured at 700 nm. A standard curve of ascorbic acid was plotted and the result was expressed as µg AAE/mg of extract.

#### Total antioxidant capacity

Total antioxidant capacity of *S. commune* (Sch1) extract was evaluated by using the standard protocol of Prieto et al. [33]. Briefly, 100 µl fungal extract was mixed with 1 ml reagent solution (28 mM sodium phosphate, 4 mM ammonium molybdate, and 0.6 M sulfuric acid). Thereafter, reaction tubes were incubated at 95℃ for 90 min. After incubation, reaction tubes were cooled to room temperature and absorbance was measured at 695 nm. Blank contained 100 µl methanol and 1 ml reagent solution. Results were expressed as µg µg AAE/mg of extract.

#### DNA damage protection assay

The potential of *S. commune* (Sch1) extract to protect the DNA damage was assessed according to the method described by Lee et al. [70]. In this assay, 1 µl pBR 322 plasmid DNA (100 µg/100µl) was mixed with 10 µl Fenton’s reagent (30 mM hydrogen peroxide, 50 µM ascorbic acid, and 80 µM FeCl_3_) followed by addition of different concentrations (2.5 µg, 5 µg, 7.5 µg, and 10 µg) of fungal extract. Distilled water was added to make the final volume upto 20 µl. Rutin was used as positive control. Reaction mixture was incubated for 30 min at room temperature. Analysis of DNA was performed using agarose gel electrophoresis.

#### CBMN assay on lymphocytes

CBMN assay for the evaluation of genoprotective potential of *S. commune* (Sch1) extract was conducted as explained by Fenech et al. [71] with some modifications. Briefly, in 5ml fully supplemented Hi-Karyo XL Roswell Park Memorial Institute culture medium 400 µl of whole blood was added. DNA damaged was induced via addition of 250µM hydrogen peroxide followed by exposure to different concentrations of fungal extract (100 µg/ml, 250 µg/ml, 500 µg/ml) and cultures were incubated at 37℃. To block cytokinesis, 4.5 µg/ml cytochalasin-B was added after 44 h of incubation. After 72 h, cultures were harvested and slides were prepared [72, 73]. From each culture 500 well spread cells were scored for the detection of micronuclei and other nuclear abnormalities including binucleated cells, nuclear bud and nucleoplasmic bridge.

### Determination of *in vivo* antioxidant and genoprotective potential

#### Experimental design

Fresh water fish *Channa punctatus* (28-32 g; 12-14.5 cm) was procured from local market, and acclimatized for two weeks in lab conditions in glass aquaria. Fish were fed with boiled eggs once a day before performing the experiment. Subsequently, water of the aquaria was replaced on daily basis to remove debris and excretory materials. Fish were randomly divided into four different groups with three fish each. BPA was used to induce oxidative stress and genotoxicity. A sub lethal dose (3.81 mg/l), half of LC_50_ determined in previous study conducted in our lab [47] was used. Group I: normal healthy control; Group II: BPA; Group III: BPA + *S. commune* (Sch1) extract (5 mg/100 g body weight); Group IV: BPA + *S. commune* extract (10 mg/100 g body weight). *S. commune* (Sch1) extract used in this study was dissolved in phosphate buffer saline (pH 7.4) and a single dose of the extract was administered at the beginning of the experiment. Effect of the *S. commune* (Sch1) extract on fish exposed to BPA was determined after 24, 48, 72 and 96 h using comet assay, micronuclei assay, and by estimating activity of CAT, SOD, and GR. On sampling day, blood was collected by direct puncture in the heart and stored in ethylenediamine tetraacetic acid (EDTA) coated vials to perform micronuclei assay and comet assay. Thereafter, fish were euthanized (by decapitation) and different organs including liver, kidney, and gill were collected.

#### Micronuclei assay

A small drop of blood sample was placed on glass slide and a thin smear was prepared. Thereafter, slides were fixed in absolute methanol for 20 min, air dried and subjected to Giemsa staining for 25–30 min. After this slides were air dried and observed under light microscope (Olympus CX21, Tokyo, Japan). A total of 100 cells were scored for each blood sample.

#### Comet assay

Comet assay was conducted by using the standard protocol described by Ahuja and Saran [74] with minor modifications. Microscopic glass slides were coated with 1% normal melting point agarose and dried overnight at 37℃. Blood sample (10 µl) was mixed with phosphate buffer saline (pH 7.4) to make 1ml final volume. A second layer of well mixed 85 µl low melting point agarose (0.5%) and 20 µl diluted blood was applied on the precoated glass slides, covered with cover slips and incubated at 4℃ for 15–20 min. Following incubation, gentle removal of coverslips and pouring of third layer of 0.5% low melting point agarose was done and slides were incubated again at 4℃ for 15 min and afterwards placed in lysing solution for 2.5 h. After the lysing step, slides were placed in electrophoretic buffer (pH 13) for 20 min followed by electrophoresis at 300mA and 25 V for 20 min. Subsequently, slides were coated drop wise with neutralization buffer for 10 min, buffer was drained and neutralization was performed thrice. Slides were dried overnight after washing with distilled water. Dried slides were stained with ethidium bromide and observed under fluorescent microscope (40X). Excitation filter of 515–560 nm with barrier filter of 590 nm was used. Analysis of images for the determination of comet in blood cells (100cells/sample) was performed by using CaspLab software. Comet tail length (TL) and tail intensity (% tail DNA) were selected as standard parameters for cell scoring.

### Estimation of antioxidant enzymes

#### CAT estimation

CAT activity was measured by following the method described by Aebi [75] with some modifications. Liver homogenate was prepared in 0.05 M potassium phosphate buffer (pH 7.0). Briefly, reaction mixture consisted of 2.95 ml of 20 mM hydrogen peroxide (freshly prepared in 0.05 M potassium phosphate buffer) and 0.05 ml sample. The change in the absorbance was recorded at 240 nm at 25℃. Catalase activity was expressed as mM/min/mg protein.

#### SOD estimation

Estimation of SOD in sample was performed by using method of Kono [76]. Liver homogenate was prepared in 50 mM sodium carbonate buffer (pH 10.0). The reaction mixture comprised 0.650 ml sodium carbonate buffer (50 mM, pH 10.0), 0.250 ml NBT, 0.05 ml triton X-100, 0.05 ml hydroxylamine hydrochloride (20 mM), and 0.250 ml sample. The change in the absorbance was recorded at 560 nm at 30℃. Activity of SOD was expressed as units/mg protein.

#### GR estimation

GR activity was assayed by method of Carlberg and Mannervik [77]. For this assay liver homogenate was prepared in 0.1 mM potassium phosphate buffer (pH 7.6). The reaction mixture contained 0.650 ml of 0.1 mM potassium phosphate buffer, 0.1 ml EDTA (3 mM), 0.1 ml NADPH (0.1 mM in 10 mM Tris-Hcl, pH 7.0), 0.1 ml oxidized glutathione and 0.05 ml sample. The change in the absorbance was recorded at 340 nm at 30℃. GR activity was expressed as µM/min/mg protein.

#### Histopathology

For histopathological studies, a small section of renal, liver, and gill were collected and fixed in 10% neutral buffered formalin solution followed by dehydration of tissue in graded series of alcohol. Dehydrated tissues were embedded in paraffin wax, cut in 5 μm thick sections and stained with haematoxylin and eosin (H&E) stain to study histopathological changes under light microscope.

#### GC-MS analysis

To identify the compounds in ethyl acetate extract GC-MS analysis was performed using THERMO Trace 1300GC coupled with THERMO TSQ8000 Triple Quadrupole MS (column BP-5MS). The instrument was firstly set to an initial temperature of 50 °C followed by raise in temperature up to 200 °C, at a rate of 10 °C/min with a holding time of 5 min. Further, the temperature was increased to 260 °C at a rate of 10 °C/min with a holding time of 5 min. The injection port temperature was maintained at 260 °C and the following parameters, flow rate of helium gas at 1.0 ml/minute, ionization voltage of 70 ev, 15:1 split ratio and mass spectral scans range of 35–650 (m/z) were used. Compounds were identified by using computer searches on a NIST Ver.2.1 MS data repository and by comparison of the spectra acquired using GC–MS.

#### Statistical analysis

Graphpad prism software version 7.0 was used for statistical analysis. All the findings were expressed as mean ± standard deviation. One way ANOVA, followed by Tukey’s multiple comparisons test was used to determine the significant difference between the groups. Results with *p-*value less than 0.05 (*p* < 0.05) were considered statistically significant. Probit analysis was used to calculate IC_50_ values.

## Supplementary Information

**Additional file 1:** **Fig. S1** DNA protection potential of *S. commune* (Sch1) extract. Lane 1: plasmid DNA; lane 2: plasmid DNA + Fenton’s reagent; Lane 3: plasmid DNA+ Fenton reagent + rutin (positive control); Lane 4-7: plasmid DNA + Fenton reagent + different concentrations of *S. commune* (Sch1) extract (2.5, 5, 7.5, 10 µg); Lane 8: additional sample not related to this work. Form I- supercoiled DNA; Form II- nicked (open circle) DNA; Form III- linear DNA.

## Data Availability

All data generated or analysed during this study are included in this published article.

## Abbreviations

ROS: Reactive oxygen species
RNS: Reactive nitrogen species
BPA: Bisphenol A
DPPH: 2,2-Diphenyl-1-picrylhydrazyl
ABTS: 2,2’-Azino-bis (3-ethylbenzthiazoline-6-sulphonic acid)
FRAP: Ferric reducing antioxidant power
AAE: Ascorbic acid equivalents
NBT: Nitro blue tetrazolium
PMS: Phenazine methosulphate
CBMN: Cytokinesis blocked micronuclei
CAT: Catalase
SOD: Superoxide dismutase
GR: Glutathione reductase
EDTA: Ethylenediaminetetraacetic acid
H&E: Haematoxylin and Eosin
