# Supplementary material for: An endophytic Schizophyllum commune possessing antioxidant activity exhibits genoprotective and organprotective effects in fresh water fish Channa punctatus exposed to bisphenol A
Source: BMC Microbiol. 2022 Dec 6;22:291. doi: 10.1186/s12866-022-02713-9 (PMC9724346; doi:10.1186/s12866-022-02713-9)
Supplement: Supplementary file 1 — Additional file 1: Fig. S1 DNA protection potential of S. commune (Sch1) extract. Lane 1: plasmid DNA; lane 2: plasmid DNA + Fenton’s reagent; Lane 3: plasmid DNA+ Fenton reagent + rutin (positive control); Lane 4-7: plasmid DNA + Fenton reagent + different concentrations of S. commune (Sch1) extract (2.5, 5, 7.5, 10 µg); Lane 8: additional sample not related to this work. Form I- supercoiled DNA; Form II- nicked (open circle) DNA; Form III- linear DNA. [file 12866_2022_2713_MOESM1_ESM.docx]

**Uncropped Fig. 2a**


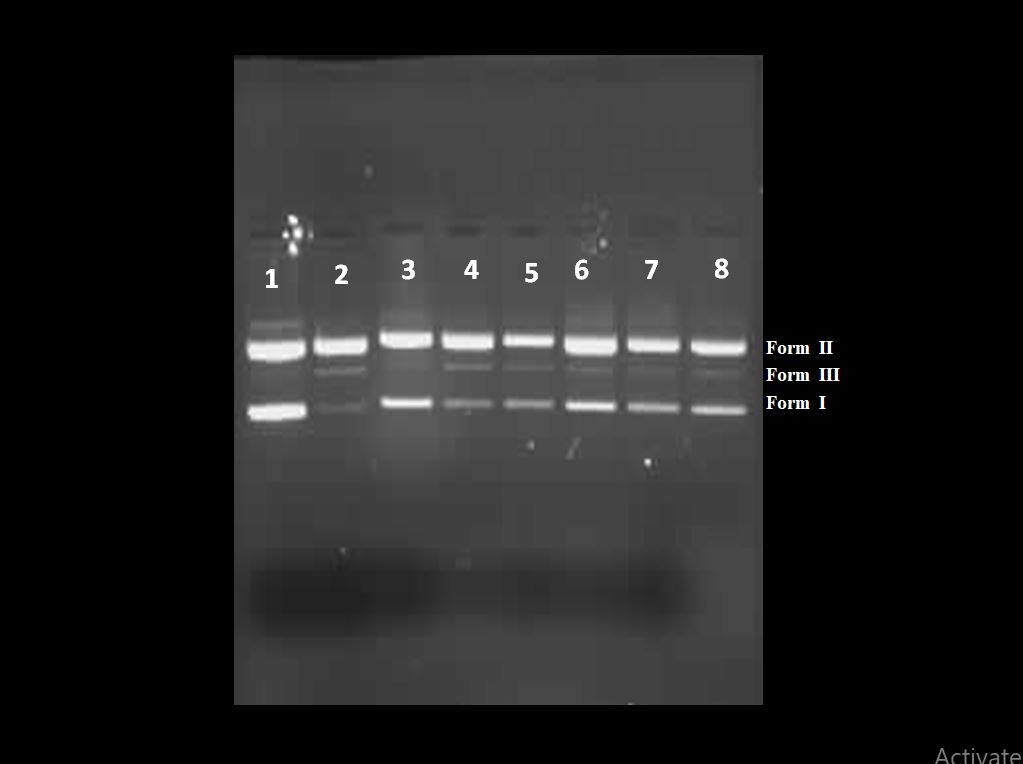


**Fig. S1** DNA protection potential of *S. commune* (Sch1) extract. Lane 1: plasmid DNA; lane 2: plasmid DNA + Fenton’s reagent; Lane 3: plasmid DNA+ Fenton reagent + rutin (positive control); Lane 4-7: plasmid DNA + Fenton reagent + different concentrations of *S. commune* (Sch1) extract (2.5, 5, 7.5, 10 µg); Lane 8: additional sample not related to this work. Form I- supercoiled DNA; Form II- nicked (open circle) DNA; Form III- linear DNA.
